# COVID-19 in Tunisia (North Africa): Seroprevalence of SARS-CoV-2 in the General Population of the Capital City Tunis

**DOI:** 10.3390/diagnostics12040971

**Published:** 2022-04-13

**Authors:** Ines Cherif, Ghassen Kharroubi, Sana Chaabane, Rihab Yazidi, Mongi Dellagi, Mohamed Ali Snoussi, Sadok Salem, Soumaya Marzouki, Wafa Kammoun Rebai, Samia Rourou, Koussay Dellagi, Mohamed Ridha Barbouche, Chaouki Benabdessalem, Melika Ben Ahmed, Jihene Bettaieb

**Affiliations:** 1 Department of Medical Epidemiology, Institut Pasteur de Tunis, University of Tunis El Manar, Tunis 1002, Tunisia; ines.cherif1993@gmail.com (I.C.); kharroubighassen@gmail.com (G.K.); chabane_sana@yahoo.fr (S.C.); rihabyazidi@yahoo.fr (R.Y.); dellagi.mongi@gmail.com (M.D.); mohamedali.snoussi@pasteur.tn (M.A.S.); sadok-salem@live.fr (S.S.); 2 Laboratory of Transmission, Control and Immunobiology of Infections (LR11IPT02), Institut Pasteur de Tunis, University of Tunis El Manar, Tunis 1002, Tunisia; marzouki_sou@yahoo.fr (S.M.); kammoun.wafa@gmail.com (W.K.R.); koussay.dellagi@pasteur.fr (K.D.); medridha.barbouche@pasteur.tn (M.R.B.); chaoukib12@gmail.com (C.B.); melika.benahmed@pasteur.tn (M.B.A.); 3 Laboratory of Molecular Microbiology, Vaccinology and Biotechnology Development, Institut Pasteur de Tunis, University of Tunis El Manar, Tunis 1002, Tunisia; samia.rourou@pasteur.tn; 4 Pasteur Network, Institut Pasteur, 75015 Paris, France

**Keywords:** SARS-CoV-2, seroepidemiologic studies, Tunisia

## Abstract

Seroprevalence studies are essential to get an accurate estimate of the actual SARS-CoV-2 diffusion within populations. We report on the findings of the first serosurvey conducted in Tunis prior to the implementation of mass vaccination and analyzed factors associated with seropositivity. A household cross sectional survey was conducted (March–April 2021) in Tunis, spanning the end of the second wave and the beginning of the third wave of COVID-19. SARS-CoV-2 specific immunoglobulin G (IgG) antibodies to the spike (S-RBD) or the nucleocapsid (N) proteins were detected by in-house ELISA tests. The survey included 1676 individuals from 431 households. The mean age and sex ratio were 43.3 ± 20.9 years and 0.6, respectively. The weighted seroprevalence of anti-N and/or anti-S-RBD IgG antibodies was equal to 38.0% (34.6–41.5). In multivariate analysis, age under 10, no tobacco use, previous diagnosis of COVID-19, a history of COVID-19 related symptoms and contact with a COVID-19 case within the household, were independently associated with higher SARS-CoV-2 seroprevalence. More than one third of people living in Tunis obtained antibodies to SARS-CoV-2. Further studies are needed to monitor changes in these figures as Tunisian population is confronted to the subsequent epidemic waves and to guide the vaccine strategy.

## 1. Introduction

The severe acute respiratory syndrome coronavirus 2 (SARS-CoV-2) was first detected in Wuhan city, China in December 2019 [1]. Since then, the virus has rapidly spread throughout the world causing 126 million cases of infection and nearly 2.8 million deaths by the end of March 2021 [2]. By mid-January 2022, the World Health Organization registered nearly 319 million cases worldwide with more than 5 million deaths [3].

In Tunisia, the first case of coronavirus disease-19 (COVID-19) was identified in 2 March 2020. The spread of infection in the country was slowed down by the strict measures imposed by the national authorities after this introduction, such as a nationwide lockdown and borders closures. As a consequence, the total number of cases and deaths registered between March and June 2020 was very modest (29 cases with no deaths and 1087 cases with 49 deaths until 17 March 2020 and 7 June 2020 respectively) [4,5]. However, after border reopening, the number of COVID-19 cases and deaths dramatically increased, shaping two COVID-19 waves: the first in August–December 2020 and the second in January–March 2021 [6]. Until 28 March 2021, 251,169 cumulative cases and 8760 COVID-19 related deaths were reported in Tunisia [7].

The monitoring of SARS-CoV-2 infection is mainly based on laboratory confirmed symptomatic cases and contacts. Hence, the true number of infected people is certainly much higher than the official reported figures mainly due to the often-asymptomatic forms of the disease. Also, some symptomatic people avoid getting tested for COVID-19 and seeking medical care for various reasons such as fear of stigma, logistical barriers or belief that COVID-19 does not exist [8,9]. A meta-analysis conducted by Chen et al. [10] at the global level included 404 serological studies published between December 2019 and December 2020 and carried out among either healthcare workers, close contacts or general population. Most of the included surveys used convenience sampling and chemiluminescence immunoassays for laboratory analysis. Based on the results of 82 seroprevalence studies of higher quality conducted among general population without known exposure to confirmed or suspected COVID-19 individuals, the estimated seroprevalence was equal to 8%. Besides, the estimated serology detected infections to confirmed cases ratio was equal to 11.1, stressing the very large burden of unreported SARS-CoV-2 infection.

Population-based seroprevalence surveys were recommended by the World Health Organization to determine as accurately as possible, the extent of the COVID-19 infection in the population [11]. Such data will provide valuable information to health authorities to tailoring prevention strategies, including vaccination.

As the extent of the SARS-CoV-2 dissemination in Tunisian communities was not sufficiently documented, we assessed the seroprevalence of SARS-CoV-2 infection among the general population in the governorate of Tunis after the second epidemic wave (from 21 March to 10 April 2021), just before the start of the vaccination campaign, and analyzed factors associated with seropositivity.

## 2. Materials and Methods

### 2.1. Study Design and Population

A cross-sectional household survey was conducted between 21 March and 10 April 2021 in anticipation of the start of mass vaccination among the general population. This period coincided with the end of the second wave and the beginning of the third epidemic wave of COVID-19 in Tunisia and was dominated by the circulation of the alpha variant [6]. The study took place in the city of Tunis in the two urban areas of El Omrane (41,781 inhabitants) and La Goulette (57,660 inhabitants) that were characterized by contrasted incidence of COVID-19 (COVID-19 incidence in El Omrane was equal to 1213 per 100,000, population which corresponds to a low to intermediate incidence and in La Goulette to 2289 per 100,000 population which corresponds to a high incidence). The two areas have similar socioeconomic characteristics, including mainly moderate-income communities.

Tunisia is located in Northern Africa at the southern shore of the Mediterranean Sea. The country population is 11.747 million according to the estimates of the National Institute of Statistics for 2020 [12] with almost 10% located in Tunis, the capital city (1,074,126 inhabitants).

The study included all persons who were permanent residents in the selected houses and who gave their consent to participate to the study. We did not include households that were unreachable after three visits of investigators. Households in which at least one member refused to participate to the survey or refused blood sampling were excluded. Nevertheless, for children younger than five years, blood test refusal was not considered as a reason for household exclusion.

### 2.2. Sampling Procedure

Households were included based on a two-stage cluster sampling. First, each study area was stratified by communities (El Omrane comprises 6 communities: France ville, Bir Atigue, Cité des oliviers, Ras Tabia, Jbel Lahmer and Oued el Sebai, and La Goulette comprises 5 communities: La Goulette, La Goulette casino, Khaireddine, Cité Essalama and Taieb El Mhiri). Within each community, a variable number of clusters of about five households proportional to its population size, were randomly selected. In each cluster, households were chosen using systematic sampling of every fifth household after a random starting point and a random direction. Then all individuals in the selected household were enrolled after being properly informed.

### 2.3. Sample Size

Assuming a design effect of 2, a prevalence equal to 50%, a precision of 0.05, a population size of 40,000, and a 95% confidence interval, the calculated sample size was equal to 760 individuals in each area, which corresponds to about 190 households with an average household size equal to 4.

### 2.4. Data Collection

In each study area, four teams of two trained investigators (one for questionnaire administration and the other for blood sampling) performed the data collection.

The face-to-face standardized questionnaire (Appendix A) included questions related to sociodemographic characteristics, lifestyle habits and medical history, compliance with barrier measures, risk factors for exposure to the SARS-CoV-2, history of COVID-19 infection and COVID-19 related symptoms.

Blood samples of about one to two milliliters were taken on a serum tube from each person. The collected sera were used for serological analysis using two in-house semi-quantitative SARS-CoV-2 ELISA tests developed and validated at Institut Pasteur in Tunis. The two tests detect immunoglobulin G (IgG) antibodies to the receptor-binding domain of the spike protein (S-RBD) or the nucleocapsid (N) proteins of the SARS-CoV-2 respectively. N and S-RBD recombinant proteins were produced in *E. coli BL21 (DE3)* and the eukaryotic expression system Sf9 respectively. ELISA assays were subsequently optimized and validated using 108 sera from RT PCR confirmed COVID-19 patients and 72 prepandemic sera from the Tunisian population collected between 2013 and 2018. Individual data are expressed as a ratio of the Optical density (OD) of the test sample to that of a reference sample selected because it gives an OD just at the level of the cut-off value of the test. This allows better comparability of the results generated by different assays by different sera.

Using a receiver operating curve, developed assays displayed have very high performances (AUC: 0.966 and 0.98, respectively, *p* < 0.0001). This resulted in a specificity of 93% and a sensitivity of 95% for the anti-S-RBD test and a specificity of 93% and a sensitivity of 94% for the anti-N test [13].

### 2.5. Data Analyses

Qualitative variables were summarized in terms of frequencies and percentages and quantitative ones in terms of means and standard deviation.

In order to facilitate data interpretation, we dichotomized the responses to the four-point Likert type scale questions related to compliance with preventive measures (namely, social distancing, hand hygiene and wear of facial mask). Hence, the responses «Always» and «Often» were grouped together on the one side, and «Never» and «Occasionally» on the other side.

Contact with a COVID-19 case within the household was defined as a contact with at least one member of the household who was previously diagnosed with COVID-19 or who was tested seropositive to the SARS-CoV-2 in the present study. COVID-19 related symptoms included respiratory symptoms, fever, chills, digestive symptoms, headache, conjunctivitis, weakness, myalgia, arthralgia, anosmia, agueusia, sore throat and loss of consciousness.

The overall seroprevalence was calculated on the basis of the detection of IgG antibodies to either the S-RBD or the N proteins of the SARS-CoV-2. This crude prevalence was afterwards weighted, using the post stratification weight method, for age and sex of the population in “El Omrane” and “La Goulette” using data published in the 2014 Tunisia population and housing census [14]. The prevalence of IgG antibodies against S-RBD and N proteins were also adjusted for ELISA test performance using the following formula [15]:

$$Adjusted\;prevalence=\frac{Crude\;prevalence+Specificity-1}{Sensitivity+Specificity-1}$$

The Chi-square test for bivariate analysis and multivariable logistic regression for multivariate analysis were used to identify factors associated with SARS-CoV-2 anti-S-RBD IgG antibodies and/or anti-N IgG antibodies seropositivity prevalence.

A *p*-value ≤ 0.05 indicates statistical significance for all analyses.

## 3. Results

During this survey, 290 households were visited by investigators in each study area. In “La Goulette”, 60 were not reached at their homes after three consecutive visits of investigators and 40 were excluded given that at least one household member (aged older than 5 years) refused to participate in the survey. In “El Omrane”, 30 households were visited by investigators, each three times, with no response, and 19 had at least one member who refused to participate in the study.

Overall, 1676 individuals from 431 households (190 in “La Goulette” and 241 in “EL Omrane”) were included in the survey. Most were female (62.5%). The mean age of participants was equal to 43.3 ± 20.9 years ranging from 1 to 100 years. Nearly a quarter (25.3%) were aged 60 years and above and 22.4% were smokers.

More than half of surveyed individuals (53.9%) were either employees or students; 58.6% did not have any underlying medical conditions and majority of them lived in an independent house (90.9%) (Table 1). The mean number of persons per room was equal to 1.4 ± 0.7.

Nearly two thirds of participants (68.4%) reported that they frequently comply with preventive measures and around the third (37.2%) declared private cars as the mean of transport they usually use. More details are presented in Appendix B.

Among all included individuals, 10.1% (95% confidence interval (CI): 8.8–11.6) reported they had already been tested for SARS-CoV-2, of whom, 42.9% (35.7–50.5) tested positive. Hence, only 4.4% (3.5–5.4) of all participants knew they had been already infected by the pandemic virus.

The weighted and test-performance adjusted prevalence of IgG antibodies against the N or the S-RBD proteins were equal to 26.6% (22.9–30.8) and 25.1% (22.2–28.4) respectively. The overall weighted seroprevalence (i.e., reactivity with S-RBD and/or N) was equal to 38.0% (34.6–41.5). At the level of each study area, it was equal to 41.9% (38.0–45.9) in “El Omrane” and to 34.0% (28.5–39.9) in “La Goulette” (More details are presented in Table 2). Applying these percentages to the total population of each study area, we found that the estimated number of infected individuals in “El Omrane” and “La Goulette” were by March 2021, 34.5 and 14.8 times higher than the reported cumulative COVID-19 cases in each area respectively.

Among all seropositive participants, more than three quarter (79.2% (75.1–82.8)) were asymptomatic.

In univariate analysis, seropositivity prevalence varied significantly according to age groups (*p* < 10^−3^). Indeed, participants younger than 10 years had the highest seroprevalence of SARS-CoV-2 infection: 51.1% (35.2–66.8) still, none of them were previously diagnosed as a COVID-19 case and only 7.1% developed COVID-19 related symptoms. A higher seroprevalence at 47.9% (41.2–54.6) was also characteristic of the group of adolescents 10–20 years old.

Seropositivity was higher among participants who were in contact (i) with a COVID-19 case within the same household (OR = 2.3 (1.8–2.9)), (ii) with those who had reported any symptom compatible with a COVID-19 infection (OR = 2.0 (1.5–2.6)) and with those who were previously tested positive for COVID-19 (OR = 4.3 (2.4–7.9)). In addition, current tobacco smokers had lower SARS-CoV-2 seroprevalence than non-smokers (OR = 0.5 (0.4–0.6)) (Table 3).

In multivariate analysis, young age, previous diagnosis of COVID-19 infection, COVID-19 related symptoms, no tobacco use and contact with a COVID-19 case within the household were independently associated with SARS-CoV-2 seropositivity (Table 4).

## 4. Discussion

In the present study, we conducted a cross-sectional survey to assess the seroprevalence of SARS-CoV-2 in people living in two urban areas of Tunis: El Omrane and La Goulette. The two study areas were selected because they expressed contrasted incidence (intermediate to low versus high) of COVID-19, based on the cumulative incidence since the pandemic’s onset. The weighted prevalence of seropositivity in the study population, defined by the detection of IgG antibodies against the N and/or the S-RBD proteins, was equal to 38.0% (34.6–41.5). In multivariate analysis, we found that younger age, smoking status, previous confirmed COVID-19 infection, history of COVID-19 related symptoms, and contact with a COVID-19 case within the household were independently and significantly associated with SARS-CoV-2 seropositivity.

**Figure 1:**
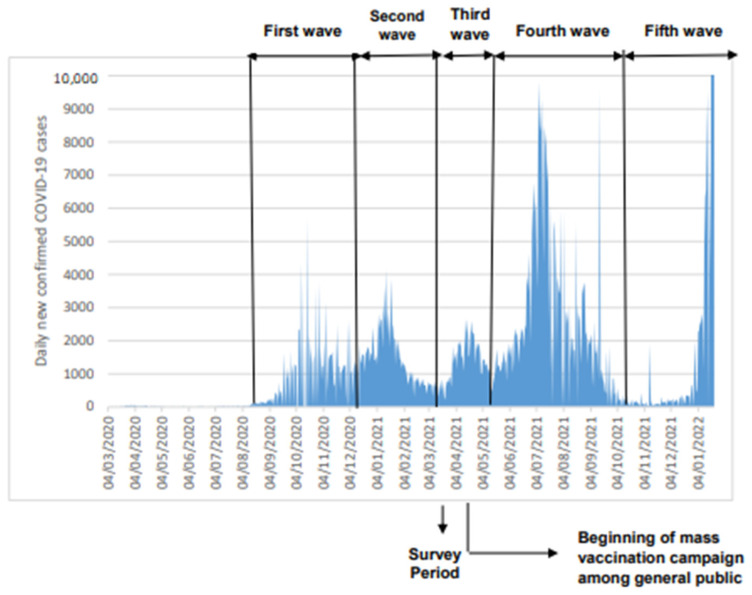
Epidemic curve COVID-19 cases in Tunisia expressed as daily new confirmed cases (Data source: [16]).

Our study was conducted on March–April 2021 corresponding to the end of the second epidemic COVID-19 wave in Tunisia and the beginning of the third one. Hence and up to the study dates, the population has been exposed mainly to the wild original SARS-CoV-2 virus and to the alpha variant. Since then, the country was hit again by two additional much higher waves: the fourth wave on May–October 2021 mainly due to the circulation of the delta virus variant [6] (Figure 1) and the fifth wave starting in January 2022 due to the emergence of the Omicron virus variant.

Our results reveal that a large fraction (almost 40%) of the population of the study areas became infected after being exposed to just the second epidemic wave. In addition, the estimated seroprevalences were 34.5 and 14.8 times greater than the reported number of confirmed COVID-19 cases in “El Omrane” and “La Goulette”, respectively. These figures stress the key role played by asymptomatic infection in SARS-CoV-2 transmission and also illustrate the limits of case detection and contact tracing in the study areas. These shortcomings likely had severely jeopardized the impacts of individual preventive measures including isolation, in term of virus circulation. The prevalence figures also illustrate the high level of infection reached after an epidemic wave that was, all in all, relatively modest compared to the fourth and fifth waves that hit the country in the following 12 months.

Surprisingly, we found that the SARS-CoV-2 seroprevalence was higher in the area of “low to intermediate” COVID-19 incidence (“El Omrane”) compared to that in high COVID-19 incidence (“La Goulette”). One possible explanation could be that “La Goulette” is a seaside city and some Tunisians were living abroad while tourists arrived in the summer of 2021 after reopening of the borders, to spend holidays there. If they became infected in Tunisia during their temporary stay, they would have been registered as cases by the regional health directorates. Still, they were not included in our survey as we only considered permanent Tunisian residents.

Our results are in keeping with those reported at the global level in population surveys conducted among unvaccinated people and before the high circulation of the delta variant, which is known to be more contagious than previous variants [17]. Seropositivity rates reported worldwide [18,19,20,21,22,23,24,25,26,27], ranged from less than 2.6% in Sierra Leone [18] to 70.0% in Iquitos (Peru) [25] (Appendix C). Such heterogeneity likely reflects differences in survey designs, dates of epidemic onset, the adherence of exposed populations to social restrictions, and individual preventive measures applied in each country [28] and the type of laboratory test used.

Our results also corroborate previous studies mainly in the African continent in which a high underdetection and/or under-reporting of COVID-19 cases was noted [18,27,29,30,31]. This could be explained by the high percentage of COVID-19 asymptomatic cases. Indeed, we found that a large majority of seropositive participants (79.2%) did not develop any kind of COVID-19 related symptoms. Such high percentage of asymptomatic COVID-19 cases was also found in some other studies [32,33]. Nevertheless, a memory bias, which can lead to an overestimation of asymptomatic forms, cannot be eliminated in this survey. Indeed, participants were asked about their symptoms since the beginning of the COVID-19 pandemic in Tunisia. Such a large gap between the true number of SARS-CoV-2 infected persons and the declared cases of COVID-19 can also be explained by limited testing, fear of the disease, infection-related stigma and, in some cases, conviction that COVID-19 does not exist [8,33,34].This emphasizes the need of amplifying testing efforts, case finding and contact tracing [35], mainly with the circulation of the new Omicron variant characterized by a very high proportion of asymptomatic cases [36]. This is key to generating accurate data on SARS-CoV-2 in Tunisia and to implement evidence based public health measures to flatten the COVID-19 curve.

In this study, we found that age was independently associated with seropositivity. Indeed, children (age < 10) had the highest percentage of IgG antibodies and the same trend is observed in the next age range (10–20). According to literature, youth are more likely to have social contact than adults [37] and may be less adherent to barrier measures such as masking, hand hygiene and social distancing [38]. Contact in schools were also found to be more physical than those in the workplace [37]. Another explanation could be that children seem to have higher and more sustainable immune responses than adults [39]. However, the findings of the present study do not support most of previous research surveys that found either a lower seroprevalence among youngest participants [30,40,41,42] or a non-significant difference according to age [33,43]. Such differences in results may be explained by the fact that most of the aforementioned studies that found a lower seroprevalence among youngest participants were conducted during the first wave of the pandemic, when the majority of schools were closed, unlike during our study, which was conducted in spring 2021 after schools’ reopening. Previous longitudinal studies have found increased SARS-CoV-2 seropositivity among children along with the overall transmission of COVID-19 [44,45,46]. In addition, with the emergence of the Omicron variant, a rise in COVID-19 pediatric cases was noted [47]. This raises the concern of the potential influence of variants’ emergence on the transmission of SARS-CoV-2 among children.

We also found that none of the seropositive participants aged under 10 years were previously diagnosed with COVID-19, which shows that the spread of SARS-CoV-2 among children and adolescents is extremely underestimated in Tunisia. Public health measures to decrease SARS-CoV-2 transmission should thus include the entire population, and not only focus on adults [35]. Non-pharmaceutical intervention, including masking, hand hygiene and ventilation of indoor settings, should also be strengthened mainly in schools [48] since those under 18 years old are still not a priority target group for COVID-19 vaccination in Tunisia.

The seroprevalence to SARS-CoV-2 did not differ significantly according to sex, in line with results of previous studies [22,43,49,50]. Moreover, our study together with previous ones [22,28,40,43,51] found a higher prevalence of antibody seropositivity among participants who report a history of COVD-19 like symptoms. Consistent with other studies [43,51], previous diagnosis of COVID-19 infection and contact with a COVID-19 case within the household were also independently and significantly associated with a higher percentage of SARS-CoV-2 IgG antibodies. However, surprisingly, we found that current tobacco smokers had lower SARS-CoV-2 seroprevalence than non-smokers. A similar result was found by Alsuwaidi et al. and Paleiron et al. [22,52]. One hypothesis is that nicotine decreases the expression of the angiotensin converting enzyme 2 (ACE 2) which is a receptor of SARS-CoV-2. Another hypothesis is that SARS-CoV-2 and nicotine compete for binding to the nicotine acetylcholine receptor (nAChR) which is possibly involved in the physiopathology of COVID-19 infection [53]. However, our results should be interpreted with caution as we conducted an observational study. Also, participants may underreport their tobacco consumption introducing a social desirability bias to the survey. Indeed, a relationship between smoking and increased risk of COVID-19 infection was underlined by a British study that used mendelian randomization analysis [54]. In addition, evidence suggests that tobacco increases the risk of severe illness and deaths due to COVID-19 [55].

Finally, a non-significant association was found between seropositivity and the used means of transport. In accordance, an online survey conducted in France assessing factors associated with a higher risk to COVID-19 contagion, found that public transport was not associated with a higher risk of virus transmission unlike restaurants and bars [56].

### Strengths and Limitations

This is the first study in Tunisia that reports the extent of the COVID-19 infection among both children and adults. As well, our study is, to the best of our knowledge, the first seroprevalence survey reported from countries in North Africa. Our study was conducted at the nadir of the second epidemic wave that peaked on 4 January 2021 and the start of the third epidemic wave that peaked on 15 April 2021 (Figure 1) [6]. Importantly it took place just before the beginning of the COVID-19 vaccination campaign in Tunisia and hence the detected antibodies could be unequivocally attributed to natural SARS-CoV-2 infection and not to vaccine administration. The results of the present study can guide policy makers in tailoring vaccination strategies and are also useful indicators/parameters (such as infection rate between age groups) that can be used for mathematical modeling in order to predict the spread of the COVID-19 pandemic, which represents a decision support tool to assist policy makers for the measures imposed and lifted. Also, the serum samples were tested using two in-house ELISA tests developed by Institut Pasteur in Tunis that detect with a high sensitivity and specificity anti-N and anti-S-RBD IgG antibodies, respectively. Indeed, anti-N IgG antibodies may appear before the anti-S-RBD [57] and the latter tends to wane at a slower rate than the anti-N antibodies [58]. In fact, Schoenhals et al. found a decrease of more than 10% in the percentage of anti-N IgG antibodies among seropositive blood donors during a three month follow up in Toamasina (Madagascar) [59]. The detection in our study of antibodies to the two viral proteins provides a better chance to detect more infected cases than when using only one antigen.

Our study has some limitations. The ELISA tests detect SARS-CoV-2 antibodies which are known to be evanescent after natural infection and vaccination [60] In fact, the antibody decay after natural infection [58] may have influenced he seroprevalence rates in our study population. Also, as we conducted a cross sectional study, the association between seropositivity and contact with a COVID-19 case within the household may be underestimated. Indeed, some contacts of infected individuals may have been contaminated, but they have not yet developed antibodies when the survey was conducted. Therefore, long term cohort longitudinal serological studies are warranted to assess the temporal dynamics of prevalence rates that integrate the opposing effects of natural antibody decay and the successive reinfections by different variants.

Our study was conducted only in the capital city Tunis. Larger populational serosurveys including other regions in Tunisia, would best describe the actual dynamics of the epidemics in the country according to the diversity of local conditions (i.e., in rural areas and in the various country eco-climatic stages, effect of transborder human movements, population density, etc.). Besides, assessing, in addition to serology, the protective virus neutralizing antibodies as well as the cellular immune responses to the COVID-19 infection, would certainly improve the estimation of the actual proportion of the population immune to SARS-CoV-2.

## 5. Conclusions

Almost 40% of participants to our serosurvey had antibodies against SARS-CoV-2 after the second epidemic wave of COVID-19 in Tunisia. This figure illustrates the true proportion of individuals who became infected and as expected, was much higher than the reported number of confirmed COVID-19 cases. Our study calls for future larger seroprevalence surveys to monitor the impact of the successive epidemic waves that hit the country as well as the effects of introducing mass vaccination to COVID-19. This will inform on changes in the fraction of immune population and its impact on the evolving SARS-CoV-2 strains.

## Figures and Tables

**Table 1:** Sociodemographic characteristics and lifestyle habits of the study population, Tunis, Tunisia.

Characteristics	El Omrane (%)	La Goulette (%)	Both (%)
Total	851 (50.8)	825 (49.2)	1676 (100.0)
Sex	*n* = 851	*n* = 825	*n* = 1676
Male	327 (38.4)	301 (36.5)	628 (37.5)
Female	524 (61.6)	524 (63.5)	1048 (62.5)
Age	*n* = 851	*n* = 825	*n* = 1676
<10	49 (5.8)	33 (4.0)	82 (4.9)
(10–20)	118 (13.9)	93 (11.3)	211 (12.6)
(20–30)	88 (10.3)	94 (11.4)	182 (10.9)
(30–40)	98 (11.5)	130 (15.8)	228 (13.6)
(40–50)	122 (14.3)	151 (18.3)	273 (16.3)
(50–60)	143 (16.8)	133 (16.1)	276 (16.5)
≥60	233 (27.4)	191 (23.2)	424 (25.3)
Occupation	*n* = 848	*n* = 823	*n* = 1671
Without professional and educational activities	441 (52.0)	330 (40.1)	771 (46.1)
Employee/student	407 (48.0)	493 (59.9)	900 (53.9)
Comorbidities	*n* = 850	*n* = 823	*n* = 1673
Yes	339 (39.9)	353 (42.9)	692 (41.4)
No	511 (60.1)	470 (57.1)	981 (58.6)
Current tobacco use (*n* = 1670)	*n* = 847	*n* = 823	*n* = 1670
Yes	185 (21.8)	189 (23.0)	374 (22.4)
No	662 (78.2)	634 (77.0)	1296 (77.6)
Dwelling type (*n* = 1642)	*n* = 849	*n* = 793	*n* = 1642
Apartment	11 (1.3)	139 (17.5)	150 (9.1)
Independent house	838 (98.7)	654 (82.5)	1492 (90.9)

**Table 2:** Prevalence of Immunoglobulin G antibodies in the governorate of Tunis Tunisia.

	El Omrane % (95% CI) *	La Goulette % (95% CI)	Both % (95% CI)
Unweighted seroprevalence			
Anti-S-RBD IgG ^†^ antibodies	32.0 (28.9–35.2)	29.8 (26.8–33.0)	30.9 (28.7–33.1)
Anti-N IgG ^§^ antibodies	33.6 (30.5–36.8)	26.3 (23.4–29.4)	30.0 (27.9–32.2)
Anti-S-RBD IgG antibodies and Anti-N IgG antibodies	24.2 (21.4–27.2)	22.2 (19.5–25.1)	23.2 (21.2–25.3)
Anti-S-RBD IgG antibodies and/or Anti-N IgG antibodies	41.5 (38.2–44.8)	33.9 (30.8–37.2)	37.8 (35.5–40.1)
Weighted seroprevalence			
Anti-S-RBD IgG antibodies	30.8 (27.3–34.5)	27.4 (23.5–31.7)	29.1 (26.5–32.0)
Anti-N IgG antibodies	34.1 (30.3–38.1)	26.2 (20.7–32.6)	30.2 (26.9–33.8)
Anti-S-RBD IgG antibodies and Anti-N IgG antibodies	23.1 (19.9–26.6)	19.7 (16.4–23.5)	21.4 (19.1–24.0)
Anti-S-RBD IgG antibodies and/or Anti-N IgG antibodies	41.9 (38.0–45.9)	34.0 (28.5–39.9)	38.0 (34.6–41.5)
The weighted and test-performance adjusted seroprevalence			
Anti-S-RBD IgG antibodies	27.0 (23.1–31.2)	23.1 (18.7–28.1)	25.1 (22.2–28.4)
Anti-N IgG antibodies	31.1 (26.8–35.7)	22.1 (15.7–29.4)	26.6 (22.9–30.8)

*: 95% Confidence Interval; ^†^: Immunoglobulin G antibodies to the receptor-binding domain of the spike protein; ^§^: Immunoglobulin G antibodies to the nucleocapsid protein.

**Table 3:** Seroprevalence to SARS-CoV-2 according to study variables Tunis, Tunisia.

Variables	N	Anti SARS-CoV-2 Positive	Seropositivity Prevalence % (95% CI) *	Weighted Seropositivity Prevalence % (95% CI)	Crude OR_c_^†^ (95% CI)	*p*-Value
Sex						NS
Male	628	236	37.6 (33.9–49.4)	39.8 (33.4–46.5)	1.1 (0.9–1.4)	
Female	1048	397	37.9 (34.9–40.9)	36.9 (33.3–40.7)	1	
Age						<10^−3^
<10	82	42	51.2 (40.6–61.7)	51.1 (35.2–66.8)	1.9 (1.2–2.9)	
(10–20)	211	101	47.9 (41.2–54.6)	46.0 (39.0–53.2]	1.4 (0.9–2.1]	
(20–30)	182	50	27.5 (21.5–34.4)	27.4 (21.4–34.4)	0.6 (0.4–0.9)	
(30–40)	228	73	32.0 (26.3–38.3)	32.2 (26.4–38.6)	0.9 (0.6–1.3)	
(40–50)	273	102	37.4 (31.8–43.2)	38.1 (32.4–44.1)	1.1 (0.7–1.6)	
(50–60)	276	108	39.1 (33.6–45.0)	37.7 (32.1–43.6)	1.1 (0.7–1.6)	
≥60	424	157	37.0 (32.6–41.7)	37.1 (32.6–41.8)	1	
Occupation						NS
Without professional and educational activities	771	293	38.0 (34.6–41.5)	39.7 (35.2–44.5)	1	
Employee/student	900	338	37.6 (34.4–40.8)	37.0 (32.5–41.8)	0.9 (0.7–1.1)	
Comorbidities						NS
Yes	692	263	38.0 (34.4–49.7)	36.5 (32.1–41.1)	0.9 (0.7–1.1)	
No	981	369	37.6 (34.6–40.7)	38.7 (34.2–43.3)	1	
Current tobacco use						<10^−3^
Yes	374	98	26.2 (22.0–30.9)	25.7 (21.2–30.8)	0.5 (0.4–0.6)	
No	1296	530	40.9 (38.2–43.6)	41.2 (37.2–45.3)	1	
Respect of preventive measures						NS
Frequently	1138	457	40.2 (37.3–43.0)	39.2 (36.1–42.4)	1	
Occasionally/ Never	525	169	32.2 (28.1–36.3)	35.7 (28.5–43.6)	0.9 (0.7–1.1)	
Travelling abroad since December, 2019						NS
Yes	12	3	25.0 (8.9–53.2)	31.1 (19.1–46.2)	0.7 (0.4–1.3)	
No	1664	630	37.9 (35.6–40.2)	38.2 (34.8–41.8)	1	
Contact with a COVID-19 case within the household						<10^−3^
Yes	1198	510	42.6 (39.8–45.4)	42.7 (38.6–47.0)	2.3 (1.8–2.9)	
No	478	123	25.7 (22.0–29.8)	24.7 (20.5–29.4)	1	
Seeking care in a health facility since the beginning of the COVID-19 pandemic in Tunisia (Mars 2020)						NS
Yes	909	347	38.2 (35.1–49.4)	36.4 (32.8–40.2)	0.9 (0.7–1.1)	
No	758	281	37.1 (33.7–40.6)	39.5 (34.1–45.3)	1	
Means of transport used						NS
Car	607	229	37.7 (33.9–41.6)	38.6 (31.9–45.7)	1.0 (0.8–1.3)	
Public transport	441	164	37.2 (32.8–49.8)	35.8 (30.4–41.5)	0.9 (0.7–1.2)	
Bicycle/motorcycle	14	3	21.4 (7.8–47.6)	22.9 (7.0–53.9)	0.5 (0.1–1.7)	
Different means of transport	176	71	40.3 (33.4–47.7)	43.1 (34.6–52.1)	1.2 (0.8–1.8)	
None	394	150	38.1 (33.4–42.9)	38.3 (32.8–44.0)	1	
Previous diagnosis of COVID-19 infection						<10^−3^
Yes	73	56	76.7 (65.8–84.9)	71.6 (58.4–81.9)	4.3 (2.4–7.9)	
No	1603	577	36.0 (33.7–38.4)	36.7 (33.2–40.3)	1	
History of COVID-19 related symptoms						<10^−3^
Yes	254	148	58.3 (52.1–64.2)	52.6 (45.5–59.6)	2.0 (1.5–2.6)	
No	1408	481	34.2 (31.7–36.7)	35.5 (31.7–39.5)	1	
Dwelling type						NS
Apartment	150	49	32.7 (25.7–40.5)	33.0 (25.2–42.0)	0.8 (0.5–1.2)	
Independent house	1492	576	38.6 (36.2–41.1)	38.8 (35.1–42.5)	1	

*: 95% Confidence Interval; ^†^: Crude Odds ratio.

**Table 4:** Predictors of seropositivity among Tunisian participants as a result of multiple logistic regression analysis.

Variables	OR_a_ *	(95% CI) ^†^	*p*-Value
Current tobacco use			
Yes	0.6	(0.5–0.8)	0.001
No	1		
Previous diagnosis of COVID-19 infection			
Yes	3.1	(1.6–5.8)	<10^−3^
No	1		
History of COVID-19 related symptoms			<10^−3^
Yes	1.8	(1.3–2.5)	
No	1		
Age			0.03
<10	1.9	(1.2–2.9)	
(10–20)	1.4	(0.9–2.1)	
(20–30)	0.6	(0.4–0.9)	
(30–40)	0.9	(0.6–1.3)	
(40–50)	1.0	(0.7–1.6)	
(50–60)	1.1	(0.7–1.6)	
≥ 60	1		
Contact with a COVID-19 case within the household			<10^−3^
Yes	2.1	(1.3–2.5)	
No	1		

*: adjusted odds ratio; ^†^: 95% Confidence Interval.

## Data Availability

The datasets used and/or analyzed during the current study are available from the corresponding author on reasonable request.

## Appendix A. Questionnaire

**Household Report Form**

Unique ID    I____I       -        I_____I      -    I___I___I___I

                    Id. area          Id. community      Id. household

**1. Data collector information**

First name–Last name	/______________________________________/
Telephone number	/_____________________________________/
Date of contact	____/____/________
Appointment date for the survey	____/____/________

**2. Household information**

Address	/__________________________________________ ___________________________________________/		
Dwelling type	□ Apartment □ Independent house		
Number of rooms	_____		
Number of household members	_____		
First and last name of the household member	Sex	Age in years (months if <5 years)	Consent
	□F □H	____ years (____ months)	□Yes □ No
	□F □H	____ years (____ months)	□Yes □ No
	□F □H	____ years (____ months)	□Yes □ No
	□F □H	____ years (____ months)	□Yes □ No
	□F □H	____ years (____ months)	□Yes □ No
	□F □H	____ years (____ months)	□Yes □ No
	□F □H	____ years (____ months)	□Yes □ No
	□F □H	____ years (____ months)	□Yes □ No
	□F □H	____ years (____ months)	□Yes □ No
	□F □H	____ years (____ months)	□Yes □ No
	□F □H	____ years (____ months)	□Yes □ No
	□F □H	____ years (____ months)	□Yes □ No
	□F □H	____ years (____ months)	□Yes □ No

**Participant Report Form**

Unique ID   I____I   -  I_____I   -       I___I___I___I  - I___I___I

                   Id. area    Id. community   Id. household  Id.  participant

**1. Data collector information**

First name–Last name	/______________________________________/
Telephone number	/_____________________________________/
Date of interview	____/____/________

**2. Participant information**

First name–Last name	I__I__I__I__I__I__I__I__I__I__I__I__I__I__I__I__I__I__I__I__I__I
Sex	□ Male □ Female
Date of birth	____/____/________
Age in years (months if <5 years)	____ years ____ months
Nationality	/______________________________________/
Telephone number	/______________________________________/
Profession/activity	□ Without activities/Retired □ Student/ Kindergarten Name and location of the school, university or kindergarten: □ Having an activity Nature of the activity/profession:…………………… Location of the work/activity:……………………………

**3. Comorbidities**

Morbid obesity (BMI> 40 kg/m^2^ if age ≥ 16 years)	□ Yes □ No □ Unknown
Cancer	□ Yes □ No □ Unknown
Diabetes	□ Yes □ No □ Unknown
HIV/other immune deficiency	□ Yes □ No □ Unknown
Heart disease	□ Yes □ No □ Unknown
Asthma	□ Yes □ No □ Unknown
Chronic respiratory disease (other than asthma)	□ Yes □ No □ Unknown
Chronic liver disease	□ Yes □ No □ Unknown
Blood disorder If yes, specify the disease:	□ Yes □ No □ Unknown
Chronic kidney disease	□ Yes □ No □ Unknown
Chronic neurological disorder	□ Yes □ No □ Unknown
Pregnancy	□ Yes □ No □ Unknown □ NA
If yes, specify:- Trimestre	□ First □ Second □ Third
- Estimated date of delivery	____/___/________
Other comorbidities	□ Yes □ No □ Unknown
If yes, specify:	

**4. Tobacco smoking**

Current tobacco smoking?	□ Yes □ No

**5. Respect of barrier measures**

Do you wear a face mask in public places?	□ Always □ Often □ Occasionally □ Never
Are you following recommended hand hygiene practices (using soap and/or hydroalcoholic solutions)?	□ Always □ Often □ Occasionally □ Never
Are you following the recommended physical distancing measures?	□ Always □ Often □ Occasionally □ Never

**6. Risk factors (exposures)**

Since December 2019, have you traveled to a foreign country?		□ Yes □ No □ Unknown
Since the begining of the COVID-19 pandemic (March 2020):		
Seeking care in a health facility	□ Yes □ No □ Unknown	
What means of transportation do you usually use?	□Car □Public transport □Bicycle/motorcycle □Different means of transport □None	
		

**7. History of Covid-19 and previous diagnostic test(s) performed:**

A. Have you been tested for COVID-19? □ Yes □ No	
If No, have you presented any symptoms suggestive of Covid-19 since the diffusion of the coronavirus in Tunisia (mars 2020)? □ Yes□ No If yes, what symptoms? ………………………………..; If you answered Yes to question A, proceed to question B. If you answered No to question A, you do not have to answer the rest of the questions.	
B. Did you get a positive test result for COVID-19 □ Yes □ No If you answered No to question B, go to question C. If you answered Yes to question B, please answer questions: a, b, c, d. a. What was (were) the type(s) and result(s) of the diagnostic test(s) performed? □RT-PCR Result:…………………. □Rapid antigen test Result:………………… □Chest scan Result …………………	
b. What was the date of COVID-19 diagnosis? c. What was the clinical form of COVID-19?	____ / ____/ ________ □Symptomatic form □Asymptomatic form
d. If you had a symptomatic form, did you experience the following symptoms?	
-Fever (≥38 °C) □ Yes □ No □ Unknown	
-Sore throa □ Yes □ No □ Unknown	
-Cough □ Yes □ No □ Unknown	
-Runny nose □ Yes □ No □ Unknown	
-Shortness of breath □ Yes □ No □ Unknown	
-Sweating □ Yes □ No □ Unknown	
-Chills □ Yes □ No □ Unknown	
-Vomiting □ Yes □ No □ Unknown	
-Nausea □ Yes □ No □ Unknown	
-Diarrhea □ Yes □ No □ Unknown	
-Abdominal pain □ Yes □ No □ Unknown	
-Chest pain □ Yes □ No □ Unknown	
-Headache □ Yes □ No □ Unknown	
-Skin rash □ Yes □ No □ Unknown	
-Conjunctivitis □ Yes □ No □ Unknown	
-Muscle ache □ Yes □ No □ Unknown	
-Joint pain □ Yes □ No □ Unknown	
-Loss of appetite □ Yes □ No □ Unknown	
-Loss of taste □ Yes □ No □ Unknown	
-Loss of smell □ Yes □ No □ Unknown	
-Nosebleed □ Yes □ No □ Unknown	
-Tiredness □ Yes □ No □ Unknown	
-Convulsions □ Yes □ No □ Unknown	
-Loss of consciousness □ Yes □ No □ Unknown	
-Other symptoms □ Yes □ No □ Unknown. If yes, specify	
C. If you have had one or more diagnostic test(s) for COVID-19 and they were all negative, specify:	
a. Why did you do the test: □ Travel abroad □ Contact with a confirmed case of COVID-19 □ Presence of signs suggestive of COVID-19, if so, specify the symptoms …………………………………………………………□ Other, Specify:………………… b. Date of the last test: ___/___/_________ c. Type of the last test: □RT-PCR □Rapid antigen test □Chest scan	
Thank you for your participation

## Appendix B. Distribution of Study Subjects According to the Risk Factors for Exposure to SARS-CoV-2, Tunis, March–April 2021

**Variables**	**N**	**%**
Respect of preventive measures (*n* = 1663)		
Frequently	1138	68.4
Occasionally/Never	525	31.6
Travelling abroad since December, 2019 (*n* = 1676)		
Yes	52	3.1
No	1624	96.9
Contact with a COVID-19 case within the household (*n* = 1676)		
Yes	1198	71.5
No	478	28.5
Seeking care in a health facility since the beginning of the COVID-19 pandemic in Tunisia (Mars 2020) (*n* = 1667)		
Yes	909	54.5
No	758	45.5
Means of transport used (*n* = 1632)		
Car	607	37.2
Public transport	441	27.0
Bicycle/motorcycle	14	0.9
Different means of transport	176	10.8
None	394	24.1

## Appendix C. Some Previous SARS-CoV-2 Seroprevalence Surveys among General Public before the Beginning of Mass Vaccination Campaigns

**Country**	**References**	**Population Size**	**Study Period**	**Antibodies**	**Antibody Detection Tests**	**Seroprevalence (%)**
France	Le Vu et al. [21]	2879	May 2020	IgG anti-S ^b^ IgG anti-N ^c^ pseudo-neutralizing antibodies	In-house test (LuLISA)	4.9
UAE ^a^	Alsuwaidi et al. [22]	8831	July August 2020	IgG anti-S	Commercial test (Diasorin)	10.4
Peru, Iquitos	Álvarez-Antonio et al. [25]	716	July 2020	IgG/IgM	Rapid test	70.0
Colombia, Monteria	Mattar et al. [26]	1368	August 2020	IgG/IgM/IgA	Commercial test (ELISA)	55.3
Israel	Reicher et al. [20]	54,357	June-Sept 2020	IgG anti-S	Commercial test (Diasorin)	4.6
South Sudan (Juba)	Wiens et al. [27]	2214	August-September 2020	IgG anti-S-RBD ^d^	Commercial test (Quantitative ELISA)	22.3
Oman	Al-Abri et al. [23]	4064	November 2020	IgG anti-S	Commercial test (Diasorin)	22.0
Sri Lanka	Jeewandara et al. [24]	2547	January 2021	IgA, IgM, IgG anti-RBD	Commercial test (Wantai SARS-CoV-2 Ab ELISA)	24.5
Sierra Leone	Barrie et al. [18]	1893	March 2021	IgG/IgM	Commercial Rapid test	2.6
Zimbabwe	Fryatt et al. [19]	2340	Feb-April 2021	IgG anti-N ^b^	Commercial test Roche Elecsys	53.0
^a^: United Arab Emirates; ^b^: Immunoglobuline G antibodies to the the spike protein; ^c^: Immunoglobuline G antibodies to the nucleocapsid protein; ^d^: Immunoglobuline G antibodies to the receptor-binding domain of the spike protein.						
